# Heme Oxygenase-1 Induction Improves Cardiac Function following Myocardial Ischemia by Reducing Oxidative Stress

**DOI:** 10.1371/journal.pone.0092246

**Published:** 2014-03-21

**Authors:** Yossi Issan, Ran Kornowski, Dan Aravot, Asher Shainberg, Michal Laniado-Schwartzman, Komal Sodhi, Nader G. Abraham, Edith Hochhauser

**Affiliations:** 1 Cardiac Research Laboratory, Felsenstein Medical Research Institute, Tel-Aviv University, Petah-Tikva, Israel; 2 Cardiology Department, Rabin Medical Center, Tel-Aviv University, Petah-Tikva, Israel; 3 Cardiac Surgery department, Rabin Medical Center, Tel-Aviv University, Petah-Tikva, Israel; 4 Faculty of Life Sciences, Bar-Ilan University, Ramat-Gan, Israel; 5 Department of Pharmacology, New York Medical College, Valhalla, New York, United States of America; 6 Department of Internal Medicine, Marshall University Joan C. Edwards School of Medicine, Huntington, West Virginia, United States of America; Virginia Commonwealth University, United States of America

## Abstract

**Background:**

Oxidative stress plays a key role in exacerbating diabetes and cardiovascular disease. Heme oxygenase-1 (HO-1), a stress response protein, is cytoprotective, but its role in post myocardial infarction (MI) and diabetes is not fully characterized. We aimed to investigate the protection and the mechanisms of HO-1 induction in cardiomyocytes subjected to hypoxia and in diabetic mice subjected to LAD ligation.

**Methods:**

In vitro: cultured cardiomyocytes were treated with cobalt-protoporphyrin (CoPP) and tin protoporphyrin (SnPP) prior to hypoxic stress. In vivo: CoPP treated streptozotocin-induced diabetic mice were subjected to LAD ligation for 2/24 h. Cardiac function, histology, biochemical damage markers and signaling pathways were measured.

**Results:**

HO-1 induction lowered release of lactate dehydrogenase (LDH) and creatine phospho kinase (CK), decreased propidium iodide staining, improved cell morphology and preserved mitochondrial membrane potential in cardiomyocytes. In diabetic mice, Fractional Shortening (FS) was lower than non-diabetic mice (35±1%vs.41±2, respectively p<0.05). CoPP-treated diabetic animals improved cardiac function (43±2% p<0.01), reduced CK, Troponin T levels and infarct size compared to non-treated diabetic mice (P<0.01, P<0.001, P<0.01 respectively). CoPP-enhanced HO-1 protein levels and reduced oxidative stress in diabetic animals, as indicated by the decrease in superoxide levels in cardiac tissues and plasma TNFα levels (p<0.05). The increased levels of HO-1 by CoPP treatment after LAD ligation led to a shift of the Bcl-2/bax ratio towards the antiapoptotic process (p<0.05). CoPP significantly increased the expression levels of pAKT and pGSK3β (p<0.05) in cardiomyocytes and in diabetic mice with MI. SnPP abolished CoPP's cardioprotective effects.

**Conclusions:**

HO-1 induction plays a role in cardioprotection against hypoxic damage in cardiomyocytes and in reducing post ischemic cardiac damage in the diabetic heart as proved by the increased levels of pAKT with a concomitant inhibition of pGSK3β leading to preserved mitochondrial membrane potential.

## Introduction

Ischemia-induced cell necrosis and apoptosis are major contributors to cardiac dysfunction in ischemic heart disease It is recognized that mitochondrial dysfunction plays a crucial role in the pathogenesis of cell death induced by myocardial ischemia [1]. The change in mitochondrial permeability has been attributed to the formation and opening of a nonspecific mitochondrial permeability transition pore (mPTP) [2], [3]. The key factor responsible for mPTP opening is mitochondrial calcium overload, especially when this is accompanied by oxidative stress, adenine nucleotide depletion, elevated phosphate concentrations, and mitochondrial depolarization [4], [5], [6].

Heme oxygenase-1 (HO-1), a stress response protein, is the rate-limiting enzyme in the catabolism of heme to free iron, carbon monoxide (CO), and biliverdin, which is rapidly converted to bilirubin. HO-1 plays an important role in cellular protection against oxidative insult in cardiovascular disease, including diabetes, and in the alleviation of vascular diseases. Current knowledge highlights the importance of HO-1, specifically within the heart, due to the action of the products of heme degradation. Excess heme, due to its pro-oxidant and pro-inflammatory properties, contributes to an increase in free radical formation and cell injury [7]. Heme degradation generates biliverdin and bilirubin, which possess potent antioxidant properties [8] and are cytoprotective in the cardiovascular system [9]. HO-1 plays an important role in maintaining redox status within the heart, thereby protecting against oxidative insult. HO-1 gene expression reverses ischemic heart injury [10] and prevents vascular dysfunction in experimental diabetes [11]. We have reported that diabetic patients have lower levels of HO-1 and elevated levels of inflammatory and oxidative stress markers [12].

HO-1 induction alters the morphology of adipocytes from a few large to many small adipocytes. These smaller adipocytes exhibit increases in AMPK and adiponectin (Acrp30), while exhibiting the decreased production of inflammatory markers and cytokines (including TNFα) [13]. Unlike most other adipokines, the plasma level of adiponectin is reduced in obesity, diabetes and related pathologies [14], [15]. In animals models, administration of recombinant adiponectin protects against almost all the major obesity-related disorders, including insulin resistance [16], hypertension [17], atherosclerosis [18], and HF [19]. Adiponectin can directly affect signaling in myocardial cells and exerts beneficial activities on the heart after pressure overload and ischemia–reperfusion injury [20], [21], [22].

In this study we elucidate the protective effects of CoPP pretreatment against hypoxic-induced cardiomyocyte damage. We also hypothesized that the induction of HO-1 in diabetic mice subjected to LAD ligation improves heart function and reduce damage. We report here that increased levels HO-1 decrease hypoxic damage in cardiomyocytes and improved heart function in diabetic mice subjected to MI. In addition, HO-1 induction increased the levels of the AKT prosurvival pathway. This was associated with GSK-3β inhibition, which preserved mitochondrial membrane potential (ΔΨm) in hypoxic stress in cardiomyocytes.

## Materials and Methods

### Animals and experimental protocol

**Figure 1:**
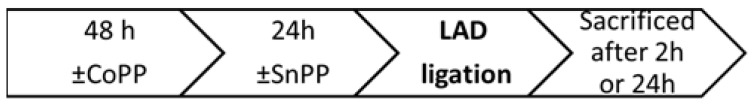
experimental protocol: Schematic illustration of the protocols used in the in vivo experiments.

All experiments were approved by the Institutional Animal Care and Use Committee at Tel Aviv University, with the Guide for the Care and Use of Laboratory Animals published by the US National Institutes of Health. Male wild type mice (C57BL) were purchased from Harlan (Jerusalem, Israel). Mice were housed in a temperature-controlled atmosphere, 12/12-hour light/dark cycle and had access to standard laboratory chow and water. The experiments were performed on 2–3 month-old male mice. CoPP (0.5 mg/100 g body) or the corresponding vehicle was given i.p. 48 h before LAD ligation. SnPP (2 mg/100 g body) or the corresponding vehicle was administered i.p. 24 h before LAD ligation. Mice were divided into 6 groups (MI and sham in each group): control mice, diabetic mice, diabetic mice + CoPP and diabetic mice + CoPP + SnPP (n = 6 mice in each group). MI or sham operation was conducted as previously described [23], using the model of left descending (LAD) coronary artery ligation. All surgery was performed under anesthesia. Mice were anesthetized (with a mixture of 100 mg/kg ketamine and 10 mg/kg xylazine) and animals were intubated and connected to a mechanical respirator. The chest was opened by a left thoracotomy and the heart was exposed. MI was induced by permanent ligation of the LAD. Sham operations were carried out by the same method but without tying the suture on the LAD. Using isoflurane anesthesia mice were sacrificed 2 h or 24 h post-surgery for blood and heart samples analyses (Fig 1). All efforts were made to minimize animals' suffering.

### Cell culture

Rat hearts (Sprague–Dawley 1–2 days old) were removed under sterile conditions and washed three times in phosphate-buffered saline (PBS) to remove excess blood cells. The hearts were minced and then gently agitated in a solution of proteolytic enzymes -RDB (Biological Institute, Ness-Ziona, Israel), which was prepared from fig tree extract. RDB was diluted 1∶100 in Ca^2+^ and Mg^2+^ free PBS for a few cycles of 10 min each, as described previously [24]. Dulbecco's modified Eagle's medium (Biological Industries, Kibbutz Beit Haemek, Israel) containing 10% horse serum was added to supernatant suspensions containing dissociated cells. The mixture was centrifuged at 300 g for 5 min. The supernatant phase was discarded, and the cells were resuspended. The suspension of the cells was diluted to 1.0×10^6^ cells/ml, and 1.5 ml of the suspension was placed in 35-mm plastic culture dishes, or on collagen/gelatin coated cover glasses. The cultures were incubated in a humidified atmosphere of 5% CO_2_ and 95% air at 37°C. Confluent monolayers exhibiting spontaneous contractions were developed in culture within 2 days. All experiments were approved by the Institutional Animal Care and Use Committee at Tel Aviv University, with the Guide for the Care and Use of Laboratory Animals published by the US National Institutes of Health.

### Hypoxic conditions

Cardiomyocyte cultures were washed in serum- and glucose-free medium before being subjected to hypoxic conditions. Exposure to argon (100%) for 120 min in a hypoxic chamber in glucose-free medium was used to simulate ischemic conditions in primary cardiac myocyte cultures. At the end of the hypoxic period, damage was evaluated using morphological and biochemical assessments.

### Experiments with CoPP and SnPP

CoPP (20 μm) or the corresponding vehicle was added to the cardiomyocyte cultures 24 h prior to hypoxia. SnPP (30 μm) or the corresponding vehicle was added 2 h after CoPP. CoPP and SnPP were purchased from Frontier Science (Logan, UT, USA) and dissolved in 0.1 M sodium citrate buffer, pH 7.8.

### Lactate dehydrogenase (LDH) and creatine kinase (CK) measurement in Cardiomyocyte culture

LDH/CK activity was determined as previously described [24]. Briefly, 25 μL growing medium was transferred into a 96-well plate and LDH/CK activity was determined with an LDH-L/CK kit (Thermo Electron, Melbourne, Australia). The product of the enzyme was measured spectrometrically at 30°C at a wavelength of 340 nm.

### Propidium iodide (PI) staining in cardiomyocyte culture

The assay is based on binding of propidium iodide (PI) to the nuclei of cells the plasma membranes of which have become permeable due to cell damage. Cell viability was determined by PI fluorometry using a multi-well plate reader (TECAN SpectraFluor Plus, Austria) and incubated for 30 min at 37°C. Fluorescence (A) from each plate/well was measured at excitation and emission wavelengths of 540 and 630 nm, respectively. The background fluorescence (B) was assessed from an unstained plate. Experiments were terminated by permeabilizing plasma membranes with 300 μM digitonin to label all nuclei with PI. A final fluorescence (C) was measured 30 min after digitonin treatment. The percentage of damaged cells (V) was calculated as V = 100(A−B)/(C−B) [25].

### Hematoxylin and eosin staining in cardiomyocyte culture

After exposure to hypoxic conditions, cardiomyocytes in the cell cultures were fixed for 10 min with 100% methanol. The fixed cells were washed twice with PBS and incubated for 15 min with hematoxylin. The stained cells were rinsed in running tap water, and stained with eosin for 15 min. The cells were then rinsed again in tap water, dehydrated and mounted on a Zeiss microscope.

### Mitochondrial membrane potential measurement

Cardiomyocyte cell cultures, grown on round coverslips, were exposed to 2-(4-(dimethylamino)styryl)-1-methylpyridinium iodide (4-di-1-ASP  =  DASPMI), dissolved in PBS (10 μmol/L) for 15 min. The coverslips were washed in PBS. The fluorescence intensity was examined under fluorescence microscopy and by a Tecan fluorimeter at 460/540 nm wavelengths. Image J software was used for quantifying DASPMI staining [26].

### Diabetes induction and monitoring

To induce diabetes in mice, we administered a single i.p. injection of 50 mg/kg streptozotocin (STZ). STZ was freshly dissolved in cold citrate buffer (pH = 4.5). Mice began fasting 6 h before STZ injection. Blood glucose level (BGL) was determined by withdrawing blood from the tail vein and measuring it with a glucose meter (Roche Accu-Chek, Basel, Switzerland).

### Two dimensional M mode echocardiography

Animals were lightly anesthetized by inhaling isoflurane. Two-dimensional (2D) guided M-mode echocardiography was performed using an echocardiogram (Siemens 512, Sequoia, U.S.A) equipped with a 15-MHz linear transducer at baseline and following LAD ligation. The 2D mode in the parasternal long-axis view was used to monitor the heart. From this view, an M mode cursor was positioned perpendicular to the interventricular septum and posterior wall of the left ventricle (LV) at the level of the papillary muscles. An M mode image was obtained at a sweep speed of 100 mm/s. Left-ventricular end-diastolic dimensions (LVEDD), and left-ventricular end-systolic chamber dimensions (LVESD) were measured. Fractional shortening (FS) was calculated as: [LVEDD – LVESD)/LVEDD]x100 (23).

### Assessment of infarct size

Midventricular viable heart sections were put in a 1% solution of 2,3, 5-triphenyl tetrazolium chloride (TTC) in phosphate buffer for 10 min at 37°C. TTC stained the viable tissue with red while the necrotic tissue remained discolored. Sections were fixed overnight in 4% formaldehyde to enhance the contrast between stained and unstained tissue. The sections were then placed between two cover slips and digitally photographed using a Nikon coolpix 5000 camera, at a resolution of 1400×960 pixels, and quantified by using ImagePro PLUS software (Media Cybernetics, USA). The area of irreversible injury (TTC-negative) is presented as a percentage of the entire area of the section [27].

### Western blotting

Frozen hearts were pulverized under liquid nitrogen and placed in a homogenization buffer (10 mmol/l phosphate buffer, 250 mmol/l sucrose, 1 mmol/l EDTA and 0.1 mmol/l phenylmethylsulfonyl fluoride (PMSF), pH 7.5). Myocytes from cell cultures were homogenized in lysis buffer and quantified for protein levels using a commercial assay (Bio-Rad, Israel). Homogenates were centrifuged at 27,000 g for 10 minutes at 4°C, the supernatant was isolated, and protein levels were visualized by immunoblotting with antibodies. Briefly, proteins (50 μg/sample) were separated using sodium dodecyl sulfate (SDS) polyacrylamide gel (12%) under denaturing conditions and electrotransferred onto nitrocellulose (Bio-Rad) for 1 h at 100 V. Membranes were blocked with 5% nonfat milk in Tris–buffered saline with 0.1% Tween 20 (TBST) for 1 h at room temperature. Primary antibodies were used at a 1∶1000 concentration in TBST with 5% nonfat milk overnight at 4°C. HO-1,AKT and BCL-2 were obtained from Cell Signaling Technology, Inc (Beverly MA, USA) and glycogen synthase kinas-beta (GSK3-β) was obtained from Santa Cruz Biotechnology (Santa Cruz, California, USA).

Immunodetection of Actin/GPDH (LI-COR Biosciences, NE, USA) was performed as an internal control. Dye 800/680 secondary antibodies were added at a concentration of 1∶15000 for 1 h at room temperature. Quantification of signals was carried out with the Odyssey program (Li CORE, Lincoln, NE, USA).

### Biochemical analysis of mice plasma

Levels of troponin T were determined in the serum using Cardiac T 2017423, Roche kit (Indianapolis, USA). Levels of CK were determined in the serum using commercial Olympus OSR6126 kit (Center Valley, PA, USA). TNF-a protein levels in the serum were assessed using ELISA semi-kits for mouse TNF-a (ELISA-Max, BioLegend, CA, USA).The high molecular weight (HMW) form of adiponectin was determined using ELISA assays (Pierce Biotechnology, Inc., Woburn, MA).

### Measurement of heart superoxide levels

Heart homogenates were placed in plastic scintillation minivials containing 5 μmol/l lucigenin for the detection of O_2_ in a final volume of 1 ml air-equilibrated Krebs solution (pH 7.4) buffered with 10 mmol HEPES-1M NaOH as described previously [28]. Lucigenin chemiluminescence was measured using a liquid scintillation counter (LS6000IC, Beckman Instruments, San Diego, CA, USA).

### Statistical analysis

Statistical differences between the mice groups were assessed by analysis of variance (ANOVA) with post hoc Bonferroni test. Data are presented as mean ±SD. Statistical significance was determined as *p*<0.05. The analyses were performed using SPSS version 15 statistical software (SPSS Inc., Chicago, IL, USA).

## Results

### The effects of HO-1 induction on cardiomyocytes exposed to hypoxia

The release of LDH and CK was used as an index of cardiomyocyte injury. Untreated hypoxic cells released increased amounts of LDH (953±16.1%) and CK (690±19.1%) relative to normoxic cells (100%), (*p* = 0.01). CoPP suppressed the release of LDH in cardiomyocytes that had undergone exposure to hypoxia. LDH levels decreased to 364±20% and CK to 209±20% of normoxia (*p* = 0.01). SnPP abolished the beneficial effect of CoPP-pretreatment and the release of LDH and CK was similar to that of the untreated hypoxic group (918±23% and 700±7% respectively, (Table 1), (*p* = 0.01). CoPP and SnPP did not cause cell damage when administered under normoxic conditions (data not shown).

**Table 1:** Measurements of biochemical markers in CoPP-pretreated cardiomyocytes subjected to hypoxia.

CK (% of normoxia level)	LDH (% of normoxia level)	
100	100	Normoxia
690±19.1^$^	953±16.1 ^$^	Hypoxia
209±20^#^	364±20^#^	Hypoxia + CoPP
700±7*	918±16.1*	Hypoxia + CoPP +SnPP

The release of LDH and CK after 2 h of hypoxia was lower in the group treated with CoPP compared with the untreated group. SnPP administration abolished the beneficial effect of CoPP pretreatment. The value of 100% was given to the level of LDH or CK released from untreated cells not subjected to hypoxia. Data are means of at least 3 replicates in 5 separate experiments. Values are means ± SD (LDH ^$^ *p*<0.01 vs. normoxia, ^#^ *p*<0.01 vs. hypoxia, *p<0.01 vs. hyp +CoPP, CK^$^ *p*<0.01 vs. normoxia, ^#^ *p*<0.01 vs. hypoxia, *p<0.01 vs. hyp +CoPP).

### The effect of HO-1 induction on cardiomyocyte viability and morphology following hypoxia

**Figure 2:**
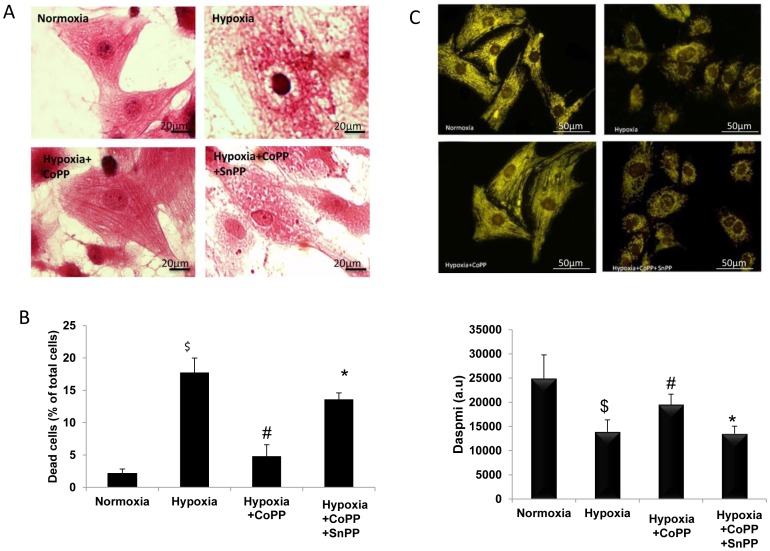
CoPP increases Cell viability and preserved mitochondrial membrane potential in cardiomyocytes culture. A: Morphology of CoPP-pretreated cardiomyocytes Treatment with CoPP preserved an improved morphological appearance of the cardiomyocytes after 2 h of hypoxia compared with the untreated group. SnPP administration abolished the beneficial effect of CoPP-pretreatment. Scale bar, 20 μm. B: The viability of CoPP-treated cardiomyocytes subjected to hypoxia Cultured cardiomyocytes were subjected to 2 h hypoxia with or without CoPP. The cells were stained with propidium iodide (PI), which marks damaged cells. The number of dead cells was lower in the group treated with CoPP compared with the untreated group. SnPP reduced the beneficial effect of CoPP-pretreatment. Data are means of at least 3 replicates in 3 separate experiments. Values are means ± SD (^$^ *p*<0.01 vs. normoxia #*p*<0.01 vs. hypoxia, *p<0.01 vs. hyp +CoPP). C: Mitochondrial function of CoPP-treated cardiomyocytes Rat cultured cardiomyocytes were subjected to 2 h hypoxic conditions with or without CoPP. D: The cells were stained with DASPMI. Hypoxic cells treated with CoPP exhibited normal mitochondrial membrane potential. SnPP administration reduced the beneficial effect of CoPP. Data are means of at least three replicates in three separate experiments. Values are means ± SD ($*p*<0.01 vs. normoxia #*p*<0.01 vs. hypoxia, *p<0.01 vs. hyp +CoPP, magnification *40).

Following 2 h of hypoxia in cardiomyocytes, hematoxylin and eosin staining demonstrated typical irreversible oncotic damage of untreated cardiomyocytes subjected to hypoxia compromising vacuoles, myofilaments disorder, and edematous areas in the cytoplasm and around the nucleus (Fig. 2A hypoxia). CoPP preserved the morphological appearance of the cardiomyocytes subjected to hypoxia, maintained symmetry of the myofilaments, retained a globular nucleus, and a clear border of the cell membrane, similar to the morphology of normoxic cells (Fig. 2A hypoxia + CoPP). SnPP administration eliminated the beneficial effect of CoPP-pretreatment resulting in cell morphology similar to that of the untreated hypoxic group (Fig 2A hypoxia + CoPP+ SnPP). Hypoxia of untreated cardiomyocytes caused cell damage of 17.7±2.2%, as revealed by PI binding, whereas under normoxic conditions PI binding was 2.14±0.55% (*p* = 0.01). CoPP significantly (*p*<0.01) increased the viability of cardiomyocytes injured by hypoxia (4.8±1.7%). SnPP abolished the beneficial effect of CoPP-pretreatment with a resultant cell damage of 15.6±0.33% hypoxic levels (Fig. 2B, *p* = 0.01.). The protective effect was also demonstrated morphologically.

### The effect of HO-1 induction on mitochondrial membrane potential in cardiomyocytes following hypoxia

In order to evaluate mitochondrial function following hypoxic stress cardiomyocytes were stained with DASPMI to estimate mitochondrial membrane potential. In normoxic cardiomyocytes DASPMI fluorescence was bright yellow and distributed in the cytosol. In many cells, localization of the mitochondria followed the striation pattern of the myofibrils. Under hypoxic conditions, untreated hypoxic cardiomyocytes exhibited low mitochondrial membrane potential. The intermyofibrillar mitochondrial patterns disappeared almost completely and a dim yellow fluorescence, mainly around the nucleus, remained (Fig. 2C Hypoxia). Pretreatment with CoPP before hypoxia protected the mitochondria from the loss of Δψ (Fig. 2C hypoxia + CoPP). This protective effect on the mitochondrial membrane potential was blocked by SnPP. Results of microfluorometric quantification of DASPMI staining are shown in Fig. 2C.

### The effect of CoPP treatment on the expression of endogenous HO-1 and AKT pathway in cardiomyocytes following hypoxia

**Figure 3:**
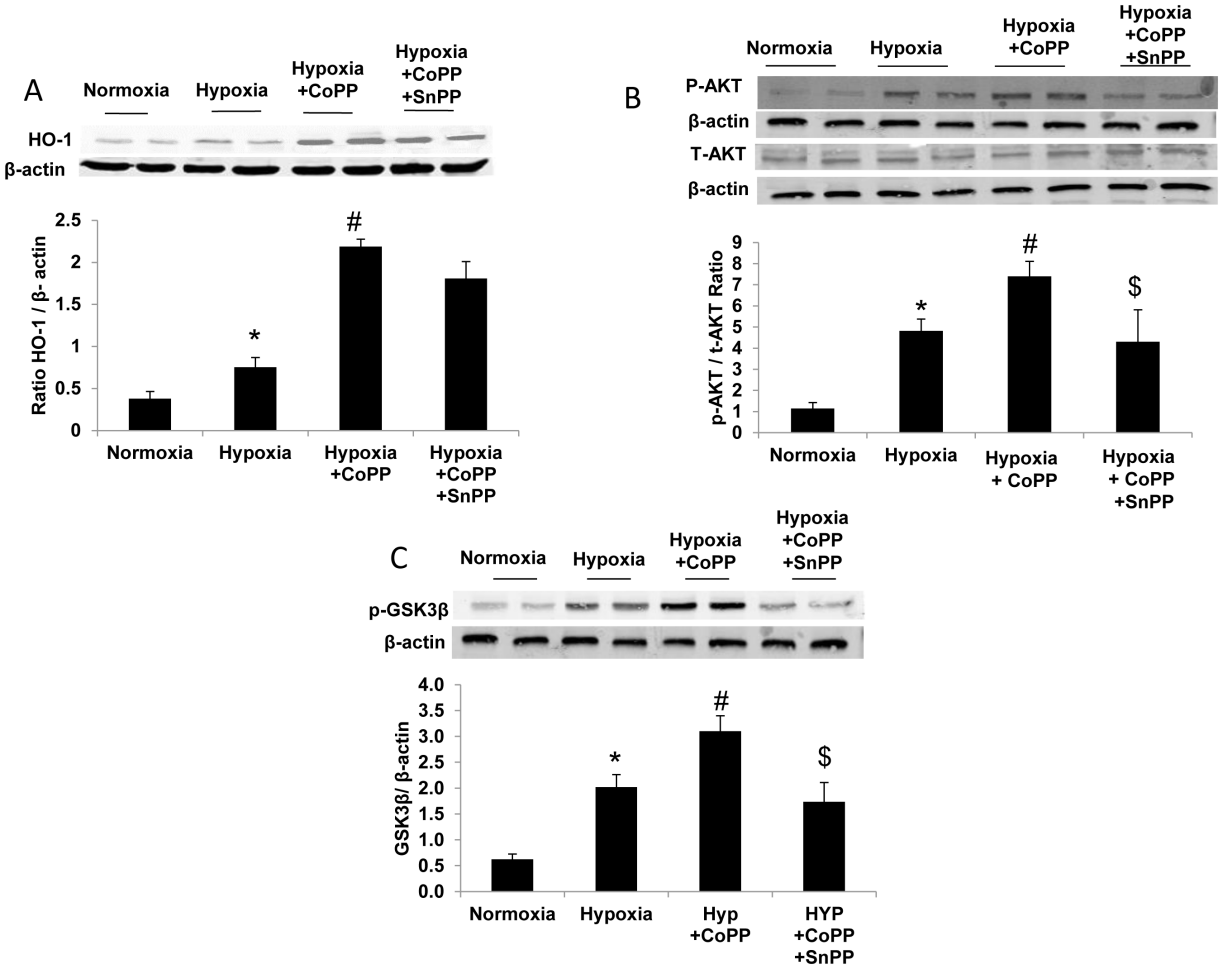
CoPP increases HO-1,AKT and GSK3β expression in cardiomyocytes culture. A: Western blot analysis of HO-1 protein in the cardiomyocyte subjected to hypoxia CoPP treatment increased HO-1 expression levels in cells subjected to hypoxia compared to non-treated cells subjected to hypoxia. Means ± SD, n = 3 in 3 different experiments (*p<0.05 vs. normoxia, ^#^ *p*<0.005 vs. hypoxia). B: Western blot analysis of AKT protein in the cardiomyocytes subjected to hypoxia: CoPP treatment increased AKT phosphorylation in cells subjected to hypoxia compared to non-treated cells subjected to hypoxia Means ± SD, n = 3 in 3 different experiments. (*p<0.01 vs. normoxia, ^#^ *p*<0.05 vs. hypoxia, ^$^ *p*<0.05 vs. hyp+CoPP). C: Western blot analysis of pGSK3β protein in the cardiomyocytes subjected to hypoxia CoPP treatment increased GSK3β phosphorylation in cells subjected to hypoxia compared to non-treated cells subjected to hypoxia. Means ± SD, n = 3 in 3 different experiments (*p<0.01 vs. normoxia, ^#^ *p*<0.01 vs. hypoxia, ^$^ *p*<0.05 vs. hyp+CoPP).

We evaluated the levels of HO-1 via Western blot analysis in response to CoPP treatment after 2 h exposure to hypoxic stress. HO-1 levels were higher in hypoxia compared to normoxia cells (*p*<0.05). CoPP increased HO-1 3-fold in hypoxia compared to the non-treated cells (*p*<0.005). SnPP did not decrease the levels of HO-1. AKT phosphorylation was increased by hypoxia compared to normoxic cells (*p*<0.01). CoPP further raised the phosphorylation of AKT compared to non-treated cells (*p*<0.05). SnPP reduced AKT phosphorylation (*p*<0.05). Total AKT (T-AKT) levels were similar in all groups Figure 3B. The elevated levels of phosphorylated AKT in the CoPP treated cells were accompanied by increased phosphorylation of its downstream target, GSK3-β (Fig. 3C). CoPP significantly (*p*<0.01) increased GSK-3β phosphorylation at Ser9 suggesting that CoPP treatment can inactivate GSK-3β in cardiac cells. Phosphorylation at this site inhibits the activation of GSK3-β thereby leading to decreased mPTP mitochondrial pores opening. SnPP reversed the action of CoPP on GSK-3β phosphorylation (Fig. 3C).

### Assessment of LV remodeling post-MI: echocardiography

**Figure 4:**
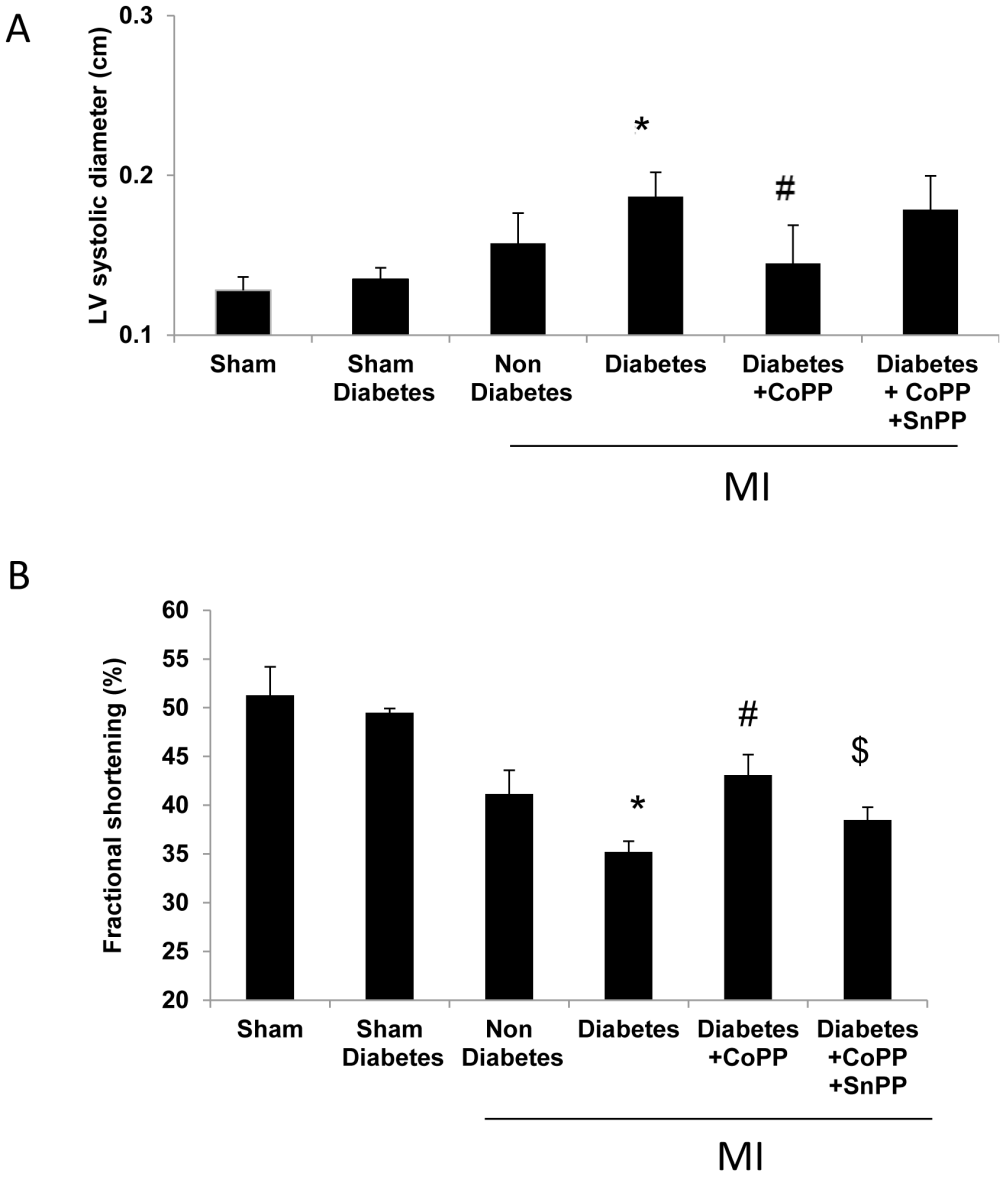
Changes in fractional shortening and left ventricle dimensions in Diabetic Mice. A: LV end-systolic diameters 24 hour post -MI. The LV end-systolic diameter increased in diabetic mice compared with non-diabetic values (*p<0 05 vs. non diabetic # *p*<0.05 vs. diabetes values are presented as means ± SD). B: Fractional shortening (FS): CoPP treatment increased FS compared to the untreated diabetic ones SnPP abolished the beneficial effect (*p<0.05 vs. non diabetic # *p*<0.01 vs. diabetes, ^$^ *p*<0.05 vs. diabetes + CoPP values are presented as means ± SD; n = 7 in control and CoPP group; n = 5 in other groups values are presented as means ± SD).

At baseline and with the sham operation, there were no differences between the groups in the echocardiographic measurements of either cardiac dimensions or function (data not shown). Induction of MI resulted in a pronounced increase of the left ventricular end-systolic diameters in the diabetic mice compared to non-diabetic mice (0.18±0.015 vs 0.15±0.018 cm respectively, *p*<0.05). This increase was reversed by CoPP treatment 48 hours before LAD ligation (0.145±0.02 cm). Adding SnPP slightly changed to 0.17±0.021 cm but it did not reach statistical significance (Fig. 4A). LV fractional shortening (FS) data are shown in Figure 4B. Diabetic mice subjected to myocardial ischemia demonstrated a significant decrease in LV FS when compared to the non-diabetic mice (35±1% vs. 41±2, respectively, *p*<0.05). CoPP increased FS in diabetic mice compared to the untreated diabetic ones (43±2%, *p*<0.01). SnPP abolished this banished effect (38.5±1.3%,).

### The effect of CoPP on cardiac damage in mice subjected to LAD ligation

**Figure 5:**
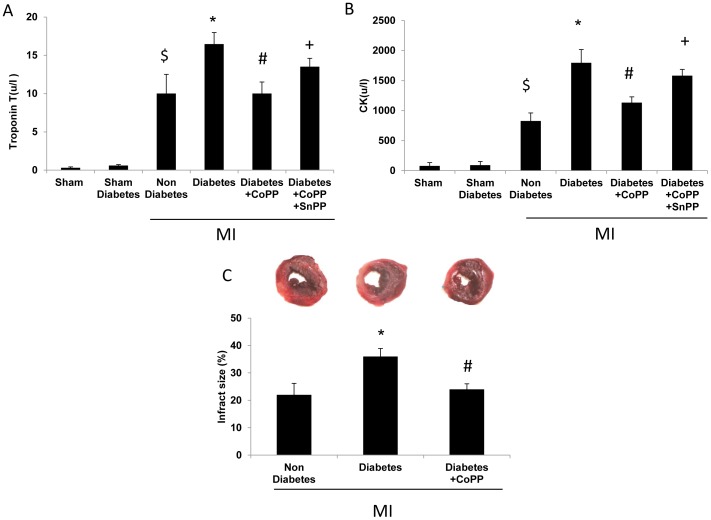
HO-1 induction reduces myocardial ischemia injury in Diabetic Mice. A, B: Measurements of biochemical markers in CoPP pretreated mice compare with untreated The release of troponin T and CK to the serum 24 h post LAD ligation was lower in the diabetic mice treated with CoPP compared with untreated diabetic mice. n = 6 mice/group Values are means ± SD (Troponin T, $ p<0.005 vs. sham non diabetes/sham diabetes,*p<0.05 vs. non diabetes MI, ^#^ *p*<0.01 vs. diabetes MI, + *p*<0.05 vs. diabetes + CoPP, CK $p<0.005 vs. Sham non diabetes/sham diabetes,*p<0.05 vs. non diabetes MI, ^#^ *p*<0.01 vs. diabetes MI, + *p*<0.05 vs diabetes +CoPP) C: Infract size by TTC staining: Hearts were isolated at 24 hours after MI and stained with TTC for the measurement of infarct area. Viable part of the heart appears red and the infarct area white. Quantification of the infarct area shows that the infarct area is significantly smaller in CoPP treated diabetic mice compared to untreated diabetic mice. Values are means ± SD; n = 6 mice/group (*p<0.05 vs. non diabetes MI, ^#^ *p*<0.05 vs. diabetes MI).

Creatine phosphokinase (CK) and cardiac Troponin T (TRP-T specific marker denoting damage in the heart muscle) were assessed. Increased levels of CK and cardiac Troponin T were detected in the serum 24 h following LAD ligation compared to sham operated mice (827.5±134 VS 80±54 u/l and 10.01±2.5 vs. 0.31±0.12 u/l respectively *p*<0.005). Diabetic mice treated with CoPP 48 h before LAD ligation exhibited lower levels of CK and Troponin T compared to non-treated diabetic mice (1134±95 vs.1797±220 u/l and 10.02±1.5 vs. 13.48±1.48 u/l respectively *p*<0.005). SnPP reversed these effects (1583±103 u/l, 13.52±1.1 u/l respectively *p* = 0.05). We also examined the effects of CoPP and STZ-diabetes on the extent of myocardial injury induced by MI. Isolated hearts were stained with TTC to identify the infarct area size. CoPP significantly reduced infarct size in diabetic mice compared to the non-treated diabetic mice, 24 h after infarction (Fig. 5C, *p*<0.05).

### The effect of CoPP on the expression of HO-1 and p-AKT in diabetic mice

**Figure 6:**
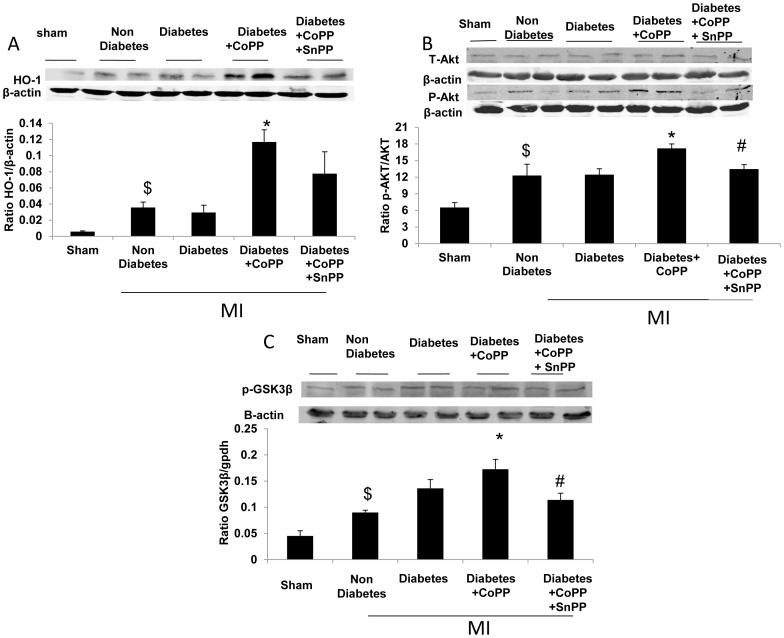
HO-1 induction increases HO-1, AKT and GSK3β expression in diabetic hearts. A:CoPP treatment significantly increased HO-1 expression levels (Means ± SD, n = 5. ^$^ *p*<0.005 vs. sham, *p<0.01 vs. diabetes,). B: Western blot analysis of AKT and p-AKT and p-Akt/Akt ratio in the different groups. CoPP treatment increased p-Akt levels while SnPP decreased the expression of p-AKT compared to the CoPP treated diabetic mice. (Means±SD, n = 5. ^$^<0.05 vs. sham,*p<0.05 vs. diabetes, #*p*<0.05 vs. diabetes+CoPP C: Western blot analysis of GSK-3β in mice subjected to LAD ligation. CoPP treatment led to an increase in GSK-3β levels; SnPP decreased GSK-3β expression compared to the CoPP treated diabetic mice (Means ± SD, n = 5. ^$^<0.05 vs. sham, * *p*<0.05 vs. diabetes, #*p*<0.05 vs. diabetes+CoPP).

Western blot analysis showed that the expression of endogenous HO-1 was lower in the sham operated compared to the MI mice (*p*<0.005). CoPP treatment increased the expression of HO-1 in diabetic mice compared to those of non-treated diabetic mice (*p*<0. 01). SnPP did not lower the levels of HO-1 (Fig. 6A). CoPP treatment 48 h before LAD ligation raised p-AKT levels compared to those of the untreated diabetic mice. Administration of SnPP 24 hours after CoPP and 24 h before LAD ligation decreased the expression of p-AKT compared to the CoPP treated diabetic mice (Fig. 6B, *p*<0.05). SnPP decreased AKT phosphorylation (*p*<0.05). The elevated levels of phosphorylated AKT in the CoPP treated mice were also accompanied by an increase in phosphorylation of GSK3-β (Fig. 6C).

### The effect of CoPP on Bcl-2 and Bax ratio in the heart of diabetic mice

**Figure 7:**
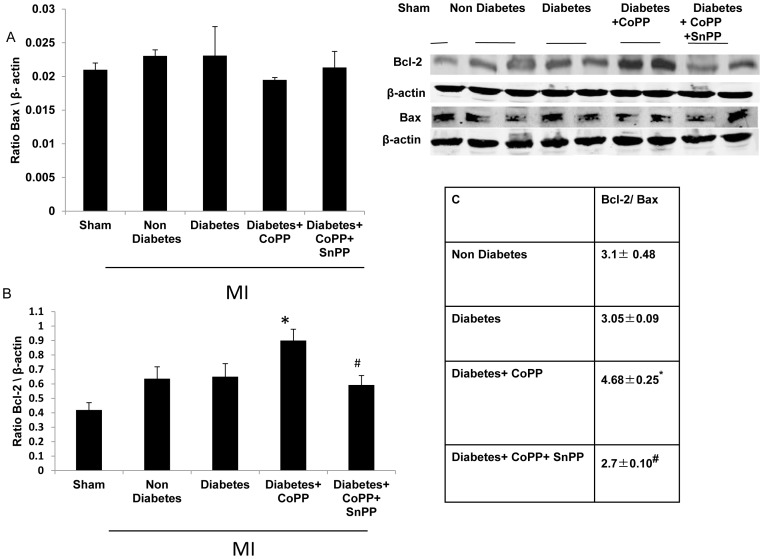
Anti-apoptotic effects of HO-1 on cardiomyocytes in diabetic mice. A: Western blot analysis of Bax B: Bcl-2 C: and Bcl-2/Bax ratio, in mice subjected to LAD ligation. CoPP treatment increased the levels of Bcl-2 expression but it did not change Bax expression to shift the Bcl-2/Bax ratio towards the antiapoptotic Bcl-2 (Means ± SD, n = 5. *p<0.05 vs. diabetes+MI, ^#^ *p*<0.05 vs. diabetes+CoPP, Table, *p<0.05 vs. diabetes+MI, ^#^ *p*<0.05 vs. diabetes+CoPP).

To capture the anti-apoptotic effect of CoPP-induced expression of HO-1 in the heart of diabetic mice, we examined the expression of survival-promoting Bcl-2. As shown in Figure 7, Western blot analysis revealed that the expression of Bcl-2 was lower in the sham group compared to the MI group. No statistical difference was noted in the expression of Bcl-2 between the non-diabetic and the diabetic mice. A higher level of Bcl-2 protein expression was detected in the CoPP treated diabetic compared to the non-treated diabetic mice. The administration of SnPP reduced the expression to levels similar to that of the untreated diabetic group. This result indicates that induced HO-1 contributes to the activation of Bcl-2. In contrast, Bax expression was similar in all the groups. CoPP treatment slightly lowered Bax expression but it did not reach statistical significance. Since Bcl-2 is an anti-apoptotic and Bax a pro-apoptotic gene, their ratio is a profound indicator of cell survival; this survival ratio can be altered either by increased Bcl-2 expression or decreased Bax expression. The increased levels of HO-1 by CoPP treatment after LAD ligation led to a shift of the Bcl-2/bax ratio towards the antiapoptotic Bcl-2. CoPP treatment increased the Bcl-2/Bax ratio by as much as 50% in the treated group compared to the untreated diabetic and control groups (4.68±0.25, 3.05±0.09, 3.1±0.48 respectively *p*<0.05).

### The effect of CoPP on oxidative stress in the heart of diabetic mice

**Figure 8:**
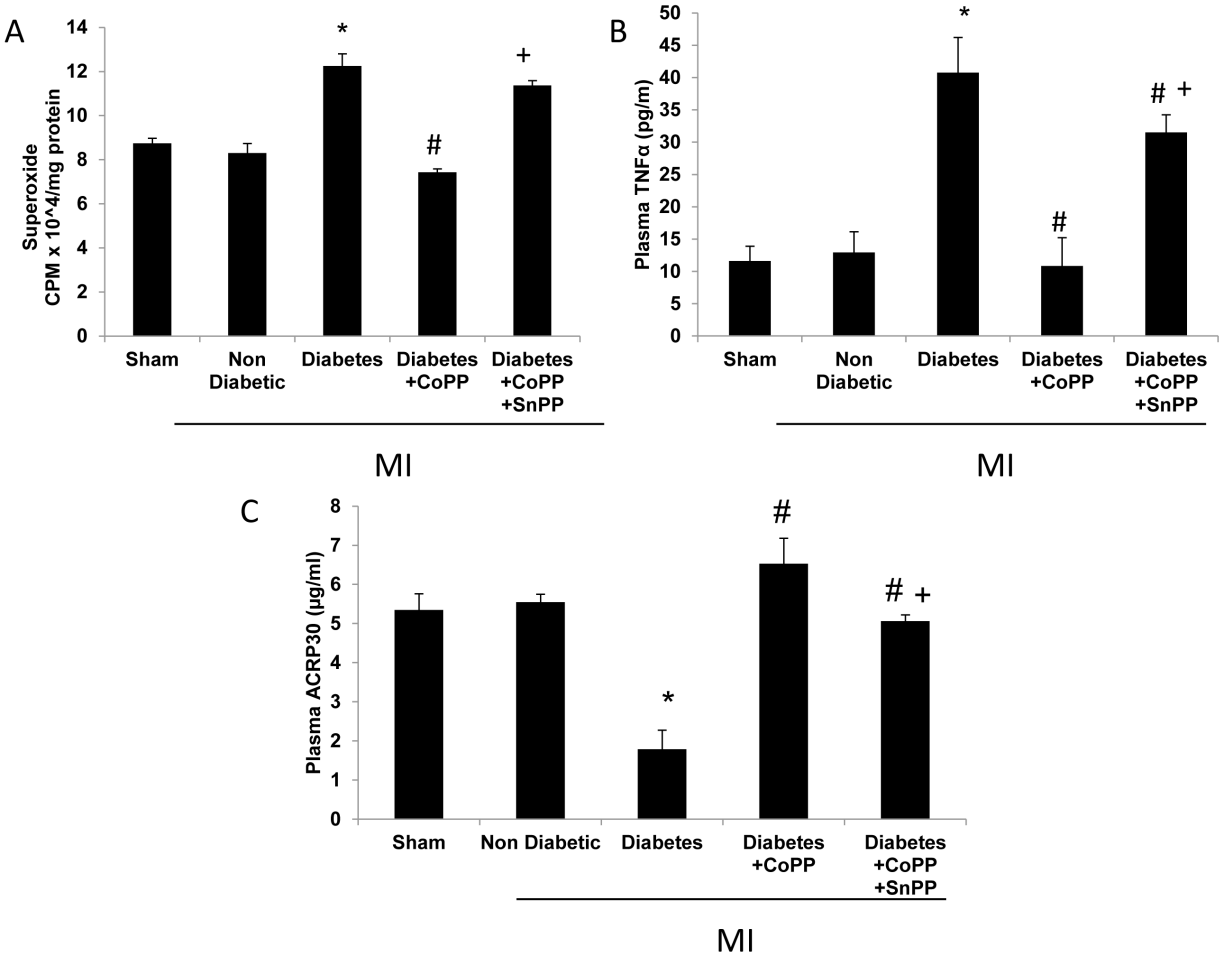
Anti-inflammatory and anti-oxidant effects of HO-1 induction on cardiomyocytes in diabetic mice. A: Superoxide levels of the cardiac tissue in mice subjected to LAD ligation. CoPP treatment increased the levels of superoxide (Means ± SD, n = 5. *<0.05 vs. non diabetes MI, #*p*<0.05 vs. diabetes MI, + *p*<0.05 vs. diabetes+CoPP. B,C: Plasma levels of TNFalpha and adiponectin respectively, in mice subjected to LAD ligation. (Means ± SD, n = 5. *<0.05 vs. non diabetes MI, #*p*<0.05 vs. diabetes MI, + *p*<0.05 vs. diabetes+CoPP MI).

Hearts from non-treated diabetic mice showed an increase in superoxide levels as compared to non-diabetic animals (12.25±0.55 vs.8.31±0.43 cpm × 10∧4/mg protein respectively, Fig. 8A, *p*<0.01). CoPP treated diabetic mice showed a decrease in superoxide levels as compared to untreated animals (7.43±0.15, *p*<0.01). SnPP increased superoxide levels to those of the diabetic groups. (11.37±0.21, *p*<0.01). TNFα levels were increased in diabetic mice compared to controls (40.76±5.4 vs.23.95±3.2 pg/ml respectively, *p*<0.05) and were decreased by CoPP (10.86±4.34 pg/ml p = 0.01). SnPP raised the TNFα levels compared to the CoPP treated diabetic mice (31.52±2.7 pg/ml p = 0.01, Fig. 8B). In contrast, diabetic mice with LAD and treated with CoPP exhibited an increase in circulating adiponectin levels compared to diabetic groups (6.53±0.65vs.1.79±0.48 μg/ml respectively). These beneficial effects were reversed by SnPP (Fig. 8C).

## Discussion

Cardiovascular disease (CVD) is a leading cause of death and disability among diabetic patients worldwide. Increased oxidative stress and inflammatory biomarkers play an important role in the initiation and progression of atherosclerotic vascular disease [29]. HO-1 plays a central role in myocardial protection from hypoxia injury. HO-1 is induced by oxidant stress and plays a crucial role in protection against oxidative insult in diabetes and cardiovascular disease [7], [30]. A potential function of HO-1 in cardiomyocytes was suggested when primary rat neonatal cardiomyocytes responded to hypoxia with increased expression levels of protein and of HO-1 mRNA, while under normal physiological conditions, HO-1 is expressed at low levels in cardiomyocytes [31]. CoPP, an inducer of HO-1, protects human cardiac stem cells against apoptosis through the activation of the ERK/Nrf2 signaling pathway [32]. HO-1 induction also enhances cell survival and restores contractility in adult cardiomyocyte grafts implanted in mice [33]. Our aim in this study was to characterize the protective capability of HO-1 induction in rat cultured cardiomyocytes exposed to hypoxic stress and in diabetic mice subjected to LAD ligation. We also wished to elucidate the possible mechanisms which provide cardioprotection. There was a direct correlation between HO-1 over expression and cardiac protection.

Our results in the in vitro study demonstrate that HO-1 induction protects cardiomyocytes against hypoxic stress. CoPP treatment in hypoxic cells reduced cell damage as measured by lower levels of LDH and CK, maintained cell morphology and increased the viability of cardiomyocytes following hypoxia, as seen by propidium iodide (PI) staining. CoPP also preserved mitochondrial membrane potential. Mitochondrial membrane potential depolarization is an early event in the intrinsic apoptotic pathway activated by hypoxia. Inhibition of HO activity by SnPP abolished the beneficial effect of CoPP-pretreatment in the cardiomyocyte culture subjected to hypoxia. Dual control mechanisms exist for metalloporphyrins as exemplified by SnPP, which potently inhibits HO activity while increasing the content of HO protein in the liver [34]. In the SnPP treated cells an increase in protein level was observed in conjunction with the abolition of the protective effects of CoPP.

Our study in mice investigated and characterized for the first time the protective capability of HO-1 induction in diabetic mice subjected to LAD ligation. This study demonstrates the beneficial effects of HO-1 after cardiac remodeling induced by MI. Because HO-1 levels are lower in diabetic patients [12] we examined the influence of HO-1 induction on damage to the heart in diabetic mice following LAD ligation. CoPP improved cardiac function as seen by increased FS, reduced infarct size and biochemical markers of myocardial injury in diabetic mice after LAD ligation. HO-1 is a rapidly inducible cytoprotective protein that degrades heme to biliverdin, ferrous iron, and carbon monoxide (CO) [8], [9]. HO-1 plays an important role in maintaining redox status within the heart, and protects the vital organ against oxidative insult. In our diabetic mice, we observed that CoPP treatment decreased oxidative stress as seen by lower levels of superoxide. HO-1 also increased the levels of adiponectin. The increase in adiponectin was associated with a significant decrease in TNF-α [13]. The dysfunction of adipose tissue, characterized by the infiltration of inflammatory cells and the aberrant production of adipokines, is a key link between obesity and cardiovascular disease [35], [36]. Adiponectin is one of the few adipokines that possesses multiple salutary effects on the prevention of cardiovascular disease [37]. Adiponectin directly affects signaling in myocardial cells and exerts beneficial activities on the heart after pressure overload and ischemia–reperfusion injury [20], [21], [22]. Adiponectin-deficient mice showed increased infarct size after ischemia-reperfusion. Exogenous adiponectin reduced infarct size in both adiponectin-deficient and wild-type mice [19].Sharma et al., showed that ischemia/reperfusion substantially enhances HO-1 expression in the porcine heart, suggesting a potential role of HO-1 in the defense against pathophysiological stress [38]. The HO-1-mediated protection from myocardial ischemia/reperfusion injury was associated with a decrease in oxidative stress and pro-apoptotic and pro-inflammatory protein levels [39]. In experimental diabetes models, overexpression of HO-1 prevented vascular dysfunction [11] and attenuated oxidative stress-dependent endothelial cell damage [40]. HO-1 overexpression promoted neovascularization and ameliorated apoptosis in a heart failure model [30]. Our study and those of others, clearly demonstrate that over expression of HO-1 in the cardiomyocyte protects against ischemia injury, thus improving cardiac function.

The mechanism(s) underlying the cardioprotective actions of HO-1 have not been fully elucidated. The cytoprotective properties of HO-1 have been attributed to the degradation of heme and the beneficial effects of its by-products, bilirubin and carbon monoxide (CO). Cells overexpressing HO-1 exhibit low levels of free iron because of the upregulation of ferritin and the extraction of iron into the extracellular space [41], [42]. The byproducts of heme degradation (bilirubin, CO, and iron as ferritin) exert actions that protect the cell from oxidative damage and death. HO-1 may also exert cytoprotective effects, independent of heme breakdown [43] by interacting with survival signaling pathways. HO-1 elevated adiponectin levels are associated with increased heart eNOS and pAKT levels, both of which increase resistance to oxidants and cell survival [10]. HO-1 improved myocardial perfusion in diabetic mice by the up-regulation of pLKB1 and AMPK signaling [44]. In the present report, we examined a novel mechanism for cardioprotective activity in cultured cardiomyocytes subjected to hypoxia. Our results indicate that an increase in AKT activity is crucial for the increased levels of HO-1 induced protection against hypoxia induced injury. CoPP treatment, in cardiomyocyte cultures before hypoxia, raised pAKT expression resulting in phosphorylation and inactivation of GSK3β (ser 9). Phosphorylation at this site inhibited the activation of GSK3-β, leading to decreased mPTP opening followed by an increase in cardiomyocyte protection [45]. Pore opening results in mitochondrial dysfunction with uncoupled oxidative phosphorylation and ATP hydrolysis, ultimately leading to cell death. Thus, mPTP is a critical therapeutic target for both pharmacological and conditional strategies to block pore formation [46]. Our results demonstrate that the protective effect that results from the induction of HO-1 is via activation of the AKT pathway, inhibition of GSK3β and protection of the mitochondria and its function in cardiomyocytes subjected to hypoxia. We hereby illustrate that in cardiomyocyte cultures CoPP preserves the mitochondrial membrane potential. Ischemic preconditioning preserves mitochondrial membrane potential and limits reactive oxygen species production in rat hearts [47]. SnPP decreased HO activity thereby preventing the activation of pAKT and the protective effect on the mitochondria seen with CoPP. The in vivo result (diabetic mice subjected to MI) supports the findings of the culture study. Our results indicate that HO-1 induction increases AKT activity in the heart of diabetic mice in a MI model; they are partly protected against hypoxia induced injury. The activation of this pathway enables cells to survive by the inhibition of proapoptotic signals and the induction of survival signals. In the treated diabetic mice AKT activation also phosphorylated and inactivated GSK3β (ser 9) inducing cardioprotection [45].

The activation of this pathway enables cells to survive by the inhibition of proapoptotic signals and by the induction of survival signals. We examined the expression of survival-promoting Bcl-2 and found a higher level of Bcl-2 protein expression in CoPP treated diabetic animals compared to the non-treated diabetic mice. This result indicates that increased HO-1 levels contribute to the activation of Bcl-2. Bcl-2 is up-regulated at the acute stage (expressed soon after the onset of infarction) suggesting that Bcl-2 protects cardiomyocytes against apoptosis. The up-regulation of Bcl-2 by CoPP in diabetic mice is important in order to limit apoptosis in the areas surrounding the infarct and to decrease damage to the heart immediately after MI.

In conclusion, we provide novel evidence that increased levels of HO-1 protects cardiomyocytes against hypoxia and reduces cardiac damage in diabetic mice subjected to LAD-ligation. HO-1 induction increases the AKT prosurvival pathway. The activation of AKT and inhibition of GSK-3β (believed to preserve the mitochondrial membrane potential (ΔΨm)) in hypoxia induces cytoprotection in cardiac myocytes and in diabetic mice with MI. Increased levels of HO-1 ameliorate apoptosis and necrotic cell death as seen by enzyme release and a decrease in the levels of apoptotic markers. Levels of adiponectin (cardioprotective) are increased while the level of the inflammatory cytokine TNFα (cardiodepressant) are decreased. Therefore, the use of either a pharmacological preconditioning agent or gene transfer to increase HO-1 levels may provide a therapeutic approach to the development of cardioprotection in humans and the enhancement of recovery and repair of the heart post-MI in CAD patients.
